# Surgical treatment of cryptorchidism: current insights and future directions

**DOI:** 10.3389/fendo.2024.1327957

**Published:** 2024-03-01

**Authors:** Niklas Pakkasjärvi, Seppo Taskinen

**Affiliations:** New Children’s Hospital, Department of Pediatric Surgery, Section of Pediatric Urology, Helsinki University Hospital, Helsinki, Finland

**Keywords:** cryptorchidism, surgical treatment, orchidopexy, laparoscopy, hormonal therapy, fertility, guidelines

## Abstract

Cryptorchidism presents with an incidence of 1-5% with potential long-term implications on future fertility and overall health. This review focuses on surgical treatment modalities, their impact on testicular development, and function while addressing the Nordic consensus statement as well as current European Association of Urology (EAU) and American Urological Association (AUA) guidelines. Congenital and acquired cryptorchidism present distinctive challenges in surgical management, with different implications for fertility. While congenital cryptorchidism entails a risk to fertility and warrants early intervention, both retractile testes and acquired cryptorchidism also pose risks to fertility potential, underscoring the importance of evaluating treatment options. Testicular location and the child’s age form the basis of a practical classification system for undescended testicles. Early diagnosis by clinical examination enables timely treatment. Imaging is reserved for selected cases only. Following guidelines, orchidopexy is recommended between 6-12 months of age for congenital cryptorchidism. Evidence increasingly suggests the benefits of early surgery for promoting testicular health and fertility potential. Current surgical options range from open to laparoscopic techniques, with the choice largely determined by the location and accessibility of the undescended testicle. The advancement in laparoscopic approaches for non-palpable testes underscores the evolving landscape of surgical treatment. Sequential surgeries may be required depending on the mobility of the undescended testes. More research is needed to explore both the potential and limitations of hormonal therapy, which is secondary to surgical treatment and can selectively have a role as adjunct to surgery. Long-term follow-up is imperative to evaluate fertility outcomes, risk of testicular malignancy, and psychological impact. By integrating current guidelines with the latest evidence, this review intends to facilitate a comprehensive understanding of cryptorchidism, thereby optimizing patient management and outcomes.

## 1 Introduction

Cryptorchidism presents with an incidence of approximately 1-5% of full-term boys at birth in Western Countries and is characterized by the failure of one or both testes to descend into the scrotum (1, 2). The incidence of cryptorchidism decreases by approximately 50% during the first six months of life, attributable to the process of natural testicular descent (3). For those individuals where this natural descent does not transpire successfully, clinical intervention becomes imperative. Left unaddressed, cryptorchidism carries a heightened risk of compromised fertility and testicular cancer (4, 5). Early intervention can substantially mitigate, if not entirely negate, these risks (6, 7). While the cooler environment of the scrotum is believed essential for spermatogenesis, the precise impact of temperature remains debated (8–11). Common to all type of stem cell niches are that these contain tightly regulated milieus for the cells to propagate and differentiate accordingly (12, 13). Therefore, despite uncertainties on the precise biological mechanisms underpinning cryptorchidism and its consequences, orchidopexy remains the primary intervention.

The fertility potential in cryptorchid boys post-orchidopexy can be inferred from the germ cells per tubular cross-section ration (G/T) (14, 15). Reduced ratios indicate a heightened infertility risk, even in cases of unilateral cryptorchidism (16). Inhibin-B, produced by the Sertoli cells, serves as an indicator of seminiferous tubule function. Its serum levels not only correlate with the count of Sertoli cells in prepubertal cryptorchid testes, but also have a strong association with the G/T ratio (17–21). Recent research indicates that low serum inhibin-B levels in boys with bilateral cryptorchidism can reliably predict a compromised G/T (22). Histological data reveal that cryptorchidism can lead to bilateral damage, even in cases of unilateral disease. In patients with unilateral cryptorchidism, 70% of scrotal testes exhibited impaired transformation from Ad spermatogonia to Ap spermatogonia (23).

While hypogonadotropic hypogonadism can cause cryptorchidism, its incidence is relatively rare compared to the overall prevalence of cryptorchidism (22, 24). Precise, age-matched gonadotropin measurements during minipuberty are crucial due to significant variability, with existing studies suggesting hypergonadotropism rather than hypogonadotropism. Consequently, current guidelines recommend gonadotropin/Gonadotropin hormone releasing hormone (GNRH) treatment mainly for clinical hypogonadotropic hypogonadism, not routinely for cryptorchidism.

Congenital cryptorchidism is associated with a heightened risk of infertility, necessitating early intervention. However, recent studies highlight that retractile testes and acquired cryptorchidism also impact fertility potential, albeit to a varying degree. Treatment decisions for these conditions are influenced by factors such as testicular mobility, but the emerging evidence suggests that the fertility implications of acquired cryptorchidism may be more significant than previously understood. This is supported by findings indicating histological changes in acquired cryptorchidism similar to those observed in congenital cases, underscoring the need for careful consideration in the management of all forms of cryptorchidism to mitigate fertility risks (25–28). The prevalence rates of acquired undescended testis were found to be 1.2% at age 6 years, 2.2% at age 9 years, and 1.1% at age of 13 years in Dutch schoolboys (29). In 66% of boys with acquired cryptorchidism, the testes have been documented with normal positions previously, highlighting the importance of consistent follow-up during childhood (30).

Testicular descent during fetal development is a complex, biphasic process governed by a series of physiological changes (1). The gubernaculum remains pivotal throughout these stages, but occasionally, it can cause the epididymis to elongate. Notably, over 50% of patients with cryptorchidism exhibit abnormalities in the epididymis and the ductus deferens (31). Furthermore, testis-epididymis dissociation, observed in 45-80%, is believed to result from a persistently abnormal genital mesentery (32). Recognizing these variations is imperative for all operating surgeons for optimal outcomes.

Early recognition and surgical repair before 1 year of age remain the most important intervention to reduce the negative impact of both unilateral and bilateral cryptorchidism (4). A systematic review and meta-analysis by Allin et al. evaluated outcomes following orchidopexy for cryptorchidism before or after one year of age (33). While they found no difference in atrophy rate between early and delayed orchidopexy, early intervention was associated with greater testicular volume and more spermatogonia per tubule, suggesting better fertility potential according to data deduced from fifteen eligible studies. A retrospective cohort study by Boehme et al. found that more than half of the orchidopexies performed after the first year of life were due to acquired undescended testis (34). Hack et al., in a study from the Netherlands in 2003, postulated that acquired cryptorchidism occurs three times more frequently than primary cryptorchidism (35). In a retrospective study of over 3500 patients in Germany, 42% of orchidopexies were performed for patients between 4 and 17 years of age during 2003-2012, however, it was unclear what proportion of these were acquired (36).

One-stage orchidopexy with spermatic vessel preservation is the standard procedure for palpable inguinal testicles. However, intra-abdominal testicles are often limited by short spermatic vessels. A debate over the optimal surgical approach persists, particularly concerning one-stage and two-stage orchidopexy for intra-abdominal testes where the limited length of the spermatic cord introduces complexities. One-stage orchidopexy involves bringing the undescended testis directly into the scrotum with possible division of the spermatic vessels during a single surgery, while two-stage orchidopexy involves dividing the spermatic vessels during an initial surgery followed by bringing the testis into the scrotum during a second surgery. The staged approach was pioneered by work from Fowler and Stephens (FS) in 1959 and is the prevalent approach for addressing high intra-abdominal testes when spermatic vessel tension precludes testicular mobilization (37). However, this technique is not without its drawbacks, particularly given the ligation of spermatic vessels which may lead to potential atrophy. The documented success rates are between 80-86% (33). To optimize vascular preservation and achieve ample mobilization, alternative methodologies have been developed. Notably, the Shehata technique is predicated on the principle of traction-induced elongation (38). Research by Ellis et al. elucidated that the gubernaculum augments blood supply in concert with the established role of the spermatic vessels and vas deferens, thus underscoring the necessity for exploring novel techniques (39). Consequently, gubernaculum sparing FS orchidopexy has gained traction as a promising approach, emphasizing the dual objectives of enhancing testicular survival and preserving cremasteric collaterals. To provide an update on current concepts, we review the surgical treatment modalities while addressing the Nordic consensus statement as well as current European Association of Urology (EAU) and American Urological Association (AUA) guidelines (40–42).

## 2 Methodology

A search was conducted on PubMed on 9.8.2023 to explore surgical treatment modalities for cryptorchidism. The following search strings were used for general review: (“surgical procedures, operative” “[MeSH Terms] OR (“surgical”[All Fields] AND “procedures”[All Fields] AND “operative”[All Fields]) OR “operative surgical procedures”[All Fields] OR (“surgical”[All Fields] AND “treatment”[All Fields]) OR “surgical treatment”[All Fields]) AND (“cryptorchidic”[All Fields] OR “cryptorchidism”[MeSH Terms] OR “cryptorchidism”[All Fields] OR “cryptorchid”[All Fields] OR “cryptorchids”[All Fields]). This search returned 2933 results. To narrow down the scope due to the abundance of studies, we restricted our search to studies published from 2011 onwards, which returned 1165 results. We then screened for eligibility and focused on comparative studies, systematic reviews, and meta-analyses for research questions in specific. The titles were initially scanned for relevance, and then abstracts were assessed. This process allowed us to curate a collection of reports that focused on the surgical treatment of cryptorchidism.

Our research for this review primarily aimed to address the following questions:

- Are there recent advancements in operative techniques that call for updates to existing protocols?
- What operative techniques are currently in use for intra-abdominal testicles?
- Does hormonal therapy serve as an effective adjunctive treatment in orchidopexy?
- How are these factors considered in the EAU, AUA, and Nordic consensus guidelines?

## 3 Guideline policies for orchidopexy

Current evidence advocates for physical assessment as the primary investigative method. For patients with a non-palpable testis, an ultrasound can be beneficial if the choice of surgical approach or the decision for diagnostic laparoscopy hinges on its findings. However, imaging studies are only to be used selectively and must not delay referrals after diagnosis (43). The EAU, AUA and Nordic Consensus guidelines uniformly recommend early intervention for undescended testes between the ages of 6 to 18 months. The AUA guidelines advocate the standard inguinal approach as the primary treatment for palpable undescended testes, whereas both the EAU and Nordic Consensus guidelines also acknowledge a scrotal approach for selected cases. For non-palpable testes, both the EAU and the Nordic consensus guidelines favor laparoscopy, while the AUA guidelines propose either open or laparoscopic surgery. Regarding the choice of one-stage and two-stage approaches, the AUA guidelines indicate that the choice is at the surgeon’s discretion. This decision is influenced by location, vascular supply, and the anatomy of the surrounding structures. On the other hand, the EAU guidelines suggest that the two-stage approach might lead to reduced testicular atrophy and improved testicular mobility. The EAU, like the AUA guidelines, does not recommend hormonal therapy as the primary treatment for undescended testes. The Nordic Consensus guidelines aligns in highlighting its limited efficacy and potential adverse reactions during childhood. The Nordic guidelines do entertain the potential consideration of hormonal treatment in exceptional cases, though tangible evidence supporting its benefits either pre- or post-surgery remains elusive. All guidelines emphasize the importance of long-term follow-up.

## 4 Impact of orchidopexy timing on testicular characteristics

Cryptorchid testes frequently display morphological abnormalities and tend to be of reduced volume (43). These abnormalities can include diffuse hypoechoic patterns, microlithiasis, and an uneven surface. While testicular location might play a more significant role in atrophy than age at surgery (44), younger patients have been observed to show fewer morphological issues (25.0% vs 83.3%, p=0.05) (45). Interestingly, the mean testicular volume ratio does not differ significantly based on whether the surgery was performed before or after two years of age (45). The G/T ratio is better maintained when orchidopexy is performed early (43). Supporting this, a systematic review by Allin et al. found that early orchidopexy correlated with a larger testicular volume and a higher number of spermatogonia per tubule, suggesting a brighter fertility prognosis (33). Nonetheless, these children may still encounter fertility challenges in the future.

## 5 Palpable testes

For palpable testes, the current consensus is for primary open orchidopexy with high success rates. Two-incision inguinal orchidopexy and single scrotal incision orchidopexy show similar results of post-operative testicular retraction and atrophy, however, the single incision technique seems more suitable for lower-positioned testes with longer cords (43). While the choice of technique is surgeon-based, according to two studies from Canada and Saudi Arabia, pediatric urologists tend to favor the single-incision approach when appropriate (46, 47). In a recent systematic review and meta-analysis, Yu et al. concluded that the single-incision orchidopexy provided benefits over traditional the two-incision approach, arguing for it to be the primary choice in low palpable cryptorchidism (48).

## 6 Intra-abdominal testes

We identified four systematic reviews on two-stage procedures for intra-abdominal testes (IAT) (**Table 1**) (43, 49–51).

**Table 1:** Outcomes of direct and staged Fowler-Stephens orchidopexies.

Study	Operation	Success rate
Penson et al. (49)	One-stage FS	79%
	Two-stage FS	86%
Wayne et al. (50)	One-stage FS	80%
	Two-stage FS	85%
Gates et al. (43)	One-stage FS	86%
	Two-stage FS	91%
Tian et al. (51)	Two-stage FS	66%
	Shehata	87%

FS, Laparoscopic Fowler-Stephens orchidopexy.

Penson et al. examined treatment options for cryptorchidism through a systematic review during 1980-2012 (49). Their objective was to assess the effectiveness of hormone therapy or surgery for cryptorchidism. They identified fourteen studies addressing hormonal therapy and 26 studies addressing surgical intervention outcomes with 695 participants undergoing primary orchidopexy, 644 participants undergoing 1-stage FS, and 784 undergoing 2-stage FS orchidopexy. They concluded that hormonal therapy alone is marginally effective as compared to placebo and may have a role for some patients. Surgical treatment, however, is associated with success rates of 96.4% for primary orchidopexy and 78.7% for 1-stage Fowler-Stephens, and 86% for 2-stage FS orchidopexy. Descent rates were comparable between primary and laparoscopic interventions as were outcome rates.

Wayne et al. conducted a systematic review to address the management of intra-abdominal testes to preserve fertility during 2008-2014 (50). They identified two systematic reviews and 29 non-randomized studies. They concluded that primary orchidopexy is effective for low IAT with a success rate of 85-100% compared to 80-85% for FS orchidopexy. The two stage FS orchidopexy was associated with complication risks, including ileus, hematoma, and infection as opposed to one-stage FS to which none of those complications was associated. Long-term follow-up revealed that 83% of two-stage FS testes were scrotal at 10-17 years postoperatively.

Gates et al. performed a systematic review on treatment options for undescended testes (UDT) during 2005-2020 addressing imaging standards, medical treatment, surgical technique, timing of operation, and outcomes (43). They included 260 articles in the review and concluded that pre-operative imaging and hormonal therapy are only reserved for special circumstances. Timing of surgery was suggested to be performed before one year of age which improves both testicular growth and potential fertility as compared to later stages. One and two-stage FS had similar rates of ascent and testicular atrophy.

Tian et al. analyzed the outcomes of FS orchidopexy as compared to the Shehata technique in a systematic review and meta-analysis in four studies published during 2021-2022 with 77 and 69 participants in each group (51). In their meta-analysis, the Shehata technique had a higher overall success rate than staged FS orchidopexy, while the operation times and retraction rates were similar. However, the success rates of two-stage FS were lower (66%) than in the three other systematic reviews.

## 7 Gubernaculum sparing laparoscopic orchidopexy

Literature regarding gubernaculum sparing laparoscopic orchidopexy remains sparse. A mere six original research studies that examined this technique were identified, of which three were comparative (**Table 2**).

**Table 2:** Outcomes of staged orchidopexies with gubernaculum sparing techniques.

Study	Operation	No atrophy	No ascent
Braga et al. (52)	One-stage FS	69%	n/a
	Two-stage FS	72%	n/a
	Two-stage GSLO	99%	91%
Roy et al. (53)	All two-stage LO	92%	94%
Zhou et al. (54)	Two-stage GSLO	99%	97%
	Two-stage GSOO	98%	97%

FS, Laparoscopic Fowler-Stephens orchidopexy; GSLO, Gubernaculum sparing laparoscopic orchidopexy; GSOO, Gubernaculum sparing open orchidopexy; LO, Laparoscopic orchidopexy.

Braga et al. compared gubernaculum sparing orchidopexy to conventional laparoscopic FS to maximize testicular blood flow and decrease atrophy rates in a prospective study where treatment arms were based on surgeon preference (52). In a cohort of 212 intra-abdominal testes, conventional FS orchidopexy was performed for 46 while gubernaculum sparing laparoscopic orchidopexy on 166 patients. Atrophy was observed in 28.3% following conventional FS and 0.6% following gubernaculum sparing laparoscopic orchidopexy, p<0.01. Interestingly, the distance of the testicle from the internal inguinal ring was not a risk factor for atrophy. They concluded that the preservation of cremasteric vessels can be beneficial for outcomes.

Roy et al. retrospectively analyzed outcomes following two-stage FS orchidopexy in a cohort of 128 intra-abdominal testes, where 96 testes were available for follow-up (53). 85% had a successful outcome. Despite the absence of evident predictors of success, the authors hypothesized that gubernaculum sparing techniques could mitigate the risk of atrophy (p=0.06; OR 3; 95% CI 0.97-9.3). No risk factors regarding intraoperative findings or operative factors were identified as associated with testicular outcome.

Zhou et al. conducted a retrospective comparison between open and laparoscopic gubernaculum sparing second stage FS in a cohort of 205 patients of which 96 underwent the gubernaculum sparing procedure (54). The overall testicular atrophy rate was 1.5% and the groupwise incidence of post-operative testicular ascent was 2.8% and 3.1% respectively. No significant difference was discerned between groups, leading them to infer that gubernaculum sparing FS orchidopexy yielded commendable survival rates and outcomes, mirroring those of more invasive open surgery.

## 8 Hormonal therapy in conjunction with surgical intervention

Hormonal therapy for cryptorchidism remains controversial. The Nordic Consensus, EAU, and AUA, advise against hormone therapy in cryptorchidism. The success of human chorionic gonadotropin (hCG) or luteinizing hormone-releasing hormone (LHRH) treatments is largely contingent upon the initial testicular location; the more inferior, the higher likelihood of descent. Pertaining to cancer or infertility, evidence remains scant. Hadziselimovic et al. advocate for hormone therapy as the primary treatment for cryptorchidism as this method not only negates the need for surgery, but in non-responsive cases, is conducive to testicular anchoring and markedly decreases the risk of testicular atrophy post-surgery (15, 55). They recommend hormonal therapy for boys with cryptorchidism at high risk for infertility and azoospermia who have undergone early orchidopexy. Thorup et al. categorized bilateral cryptorchid patients based on testicular mass, testicular germ cell number and maturation of germ cells to Ad Spermatogonia, and hormone levels (20). They identified three groups: (I) primary testicular failure, (II) transient hypothalamus-pituitary-gonadal dysfunction, and (III) normal endocrine and histopathologic evaluation. It was observed that patients with temporary hypothalamic-pituitary-gonadal dysfunction might gain from post-operative hormonal therapy (20). Findings from current studies indicate that post-operative hormone therapy might be advantageous for specific groups, however, given the small sample sizes and inconsistent results, more extensive trials are needed to provide compelling recommendations (43).

Cryptorchidism appears to be more than just an irregularity in testicular positioning as suggested by the study of Hildorf et al. on hormonal findings where 11% of boys with unilateral cryptorchidism exhibited serum inhibin-B levels below the 2.5 percentile (18). This aligns with the anticipated infertility risk shown in long-term follow-up studies. Notably, 90% of these patients displayed transient hypogonadotropic hypogonadism. This could affect the development of both testes, even if one naturally descends into the scrotum and the other is surgically positioned there. For such patients, treatment with adjuvant gonadotropin-releasing hormone may be a viable consideration.

Hormone therapy is not devoid of adverse effects, with hCG reportedly inducing virilizing effects more frequently (74%) than LHRH (5.1%) (43, 56). While many of these systemic side effects are transient and tend to dissipate within six months, there are documended side effects on the testis itself, including interstitial bleeding and germ cell apoptosis which was associated with reduced testicular volume later in adulthood (56, 57).

## 9 Conclusion

Orchidopexy remains a cornerstone in pediatric urologic surgery, constantly evolving in technique and approach. For palpable cryptorchidism, open single-stage surgery is safe and established. Current evidence gently advocates for single-incision surgery for lower testicles, however, definitive superiority over two-incision surgery remains elusive. Non-palpable cryptorchidism still poses challenges. Protecting blood flow is essential, given its role in preventing testicular atrophy. The gubernaculum sparing second-stage FS along with the Shehata variant show promise, both of which warrant further studies for affirmation. In all studies, the single-stage laparoscopic orchidopexy, which involves sacrificing the gubernaculum and its associated vessels, was found to be associated with a greater risk of testicular atrophy.

The role of hormonal therapy for cryptorchidism remains controversial. While hormonal therapy may offer some therapeutic benefit, potential risks like infertility and surgical delays cannot be ignored and warrant further investigation. Hormonal assessment may soon complement orchidopexies, offering a nuanced approach to treatment.

**Figure 1:**
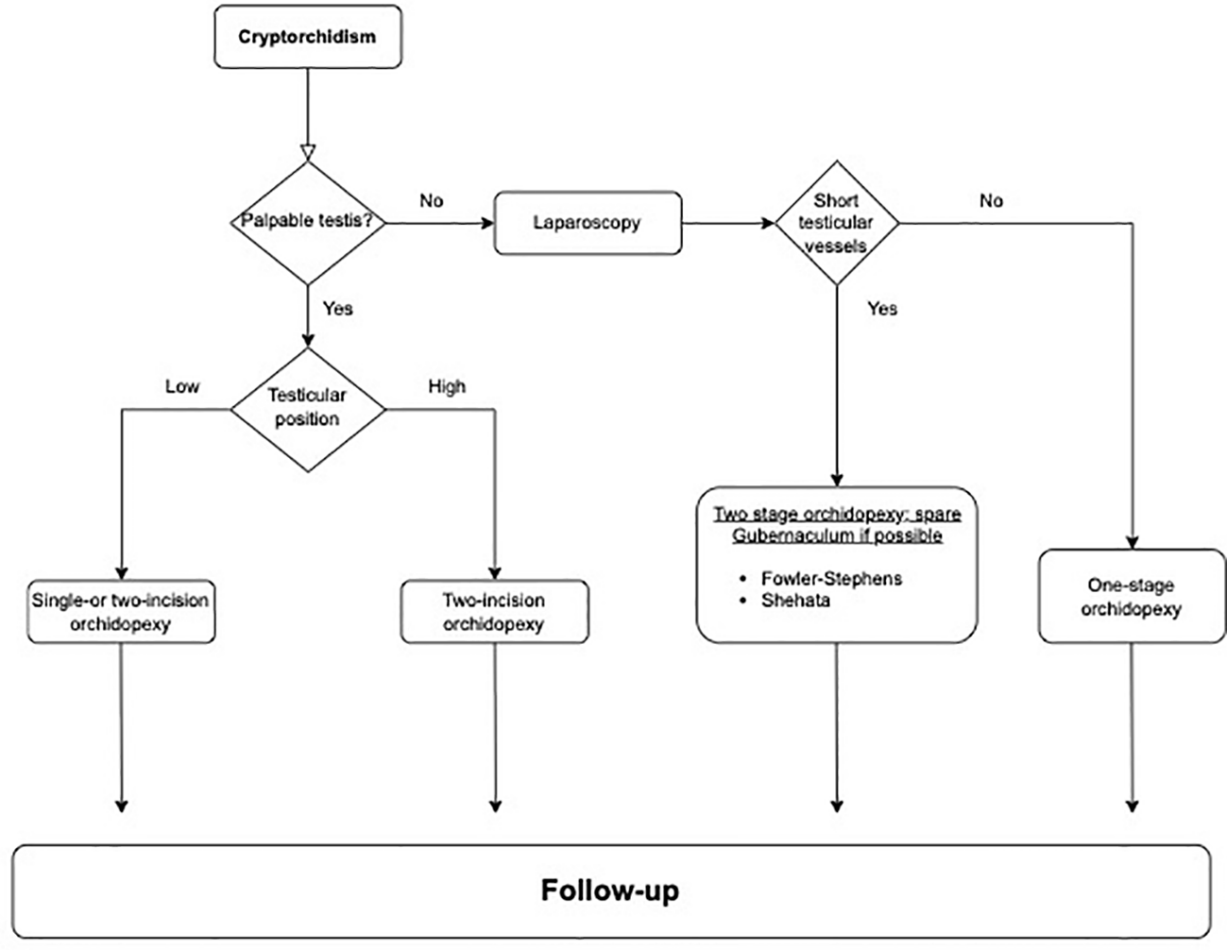
Clinical algorithm for the management of cryptorchidism.

Emerging techniques in laparoscopic orchidopexy are intriguing, but their inclusion into guidelines awaits more robust evidence. However, current treatments, aligned with existing guidelines, continue to produce excellent outcomes. Therefore, adherence to these guidelines remains paramount for best practices in patient care. To aid in applying these insights, **Figure 1** presents a proposed algorithm for managing cryptorchidism, guiding clinicians through treatment decisions from diagnosis to follow-up based on testis palpability and location. This schematic aims to streamline clinical decision-making in pediatric urologic surgery.

## Author contributions

NP: Conceptualization, Data curation, Formal analysis, Investigation, Methodology, Project administration, Resources, Software, Validation, Visualization, Writing – original draft, Writing – review & editing. ST: Conceptualization, Formal analysis, Investigation, Methodology, Project administration, Resources, Supervision, Validation, Writing – review & editing.
