# Supplementary material for: Surgical treatment of cryptorchidism: current insights and future directions
Source: Front Endocrinol (Lausanne). 2024 Mar 1;15:1327957. doi: 10.3389/fendo.2024.1327957 (PMC10940471; doi:10.3389/fendo.2024.1327957)
Supplement: Supplementary file 1 [file DataSheet_1.pdf]

| Study | Operation | Success rate |
|---|---|---|
| Penson et al.(46) | One-stage FS | 79% |
| | Two-stage FS | 86% |
| Wayne et al.(45) | One-stage FS | 80% |
| | Two-stage FS | 85% |
| Gates et al.(39) | One-stage FS | 86% |
| | Two-stage FS | 91% |
| Tian et al.(47) | Two-stage FS | 66% |
| | Shehata | 87% |
