# Supplementary material for: Surgical treatment of cryptorchidism: current insights and future directions
Source: Front Endocrinol (Lausanne). 2024 Mar 1;15:1327957. doi: 10.3389/fendo.2024.1327957 (PMC10940471; doi:10.3389/fendo.2024.1327957)
Supplement: Supplementary file 2 [file DataSheet_2.pdf]

Study	Operation	No atrophy	No ascent
Braga et al.(48)	One-stage FS	69%	n/a
	Two-stage FS	72%	n/a
	Two-stage GSLO	99%	91%
Roy et al.(49)	All two-stage LO	92%	94%
Zhou et al.(50)	Two-stage GSLO	99%	97%
	Two-stage GSOO	98%	97%
